# Metabolic Risk Susceptibility in Men Is Partially Related to Adiponectin/Leptin Ratio

**DOI:** 10.1155/2013/409679

**Published:** 2013-03-06

**Authors:** Gloria Lena Vega, Scott M. Grundy

**Affiliations:** Center for Human Nutrition and Departments of Clinical Nutrition, Internal Medicine and The Metabolic Unit of the Veterans Administration North Health Science Center at Dallas, 5323 Harry Hines Boulevard, Dallas, TX 75390-9052, USA

## Abstract

*Background*. High adiponectin/leptin ratio may be protective from metabolic risks imparted by high triglyceride, low HDL, and insulin resistance. *Methods*. This cross-sectional study examines plasma adipokine levels in 428 adult men who were subgrouped according to low (<6.5 **μ**g/mL)and high (≥6.5 **μ**g/mL)adiponectin levels or a low or high ratio of adiponectin/leptin. *Results*. Men with high adiponectin/leptin ratio had lower plasma triglyceride and higher HDL cholesterol than those with low ratio. Similarly, those with high adiponectin/leptin ratio had lower TG/HDL cholesterol ratio and HOMA2-IR than those with low ratio. In contrast, levels of adiponectin or the ratio of adiponectin/leptin did not associate with systolic blood pressure. But the ratio of adiponectin/leptin decreased progressively with the increase in the number of risk factors for metabolic syndrome. *Conclusion*. Adipokine levels may reflect adipose tissue triglyceride storage capacity and insulin sensitivity. Leptin is an index of fat mass, and adiponectin is a biomarker of triglyceride metabolism and insulin sensitivity. Men with high adiponectin/leptin ratios have better triglyceride profile and insulin sensitivity than men with a low ratio regardless of waist girth.

## 1. Introduction

Excess abdominal body fat is implicated in the etiology of the metabolic syndrome, a cluster of risk factors for cardiovascular disease and for type 2 diabetes mellitus. The risk factors include dyslipidemia (high triglyceride and/or low HDL cholesterol), insulin resistance, and hypertension [1]. Abdominal obesity is assessed by waist girth, and several cut points of high-risk waist girth have been recommended to identify at-risk individuals based on gender and ethnicity [2]. Reaching a consensus on a sex-specific, global definition of high-risk waist girth has proved to be challenging [3]. However, a global cut point may be impractical because individuals vary in susceptibility to obesity-induced risk for metabolic syndrome. An alternative to using waist girth is to identify biomarkers that are causally related to the metabolic risks and that reflect a function of adipose tissue.

Two adipokines, leptin and adiponectin, may be risk markers of fat-induced dyslipidemia and insulin resistance. Both adipokines are reportedly associated with risk for type 2 diabetes and with cardiovascular disease. Plasma levels of leptin correlate positively with total body fat [4–6] and with adipocyte number in men [7]. In addition, individuals at high risk seemingly have high levels of plasma leptin [8]. The levels are directly proportional to secretion rates of the adipokine by adipose tissue [9], which is primarily produced in subcutaneous tissue [10]. Adiponectin, in turn, is an insulin-sensitizing adipokine as well as an anti-inflammatory and antiatherogenic hormone. In obese subjects, levels of plasma adiponectin are reduced suggesting an abnormality in adipose tissue function. Furthermore, mutations in the gene encoding for adiponectin are associated with type 2 diabetes mellitus and features of metabolic syndrome including hypertension, dyslipidemia, and atherosclerosis [11]. It is still unclear whether adiponectin production differs between subcutaneous and visceral adipose tissue. However, it has been suggested that adiponectin levels in plasma are inversely correlated with visceral adiposity [12] and are positively correlated with lower extremity fat [13]. 

In the current study, levels of plasma adiponectin and leptin were examined in men with varying degrees of obesity that had marked interindividual variation in plasma triglycerides and insulin sensitivity. The question addressed was whether adiponectin alone or in combination with leptin had an effect on dyslipidemia and insulin resistance.

## 2. Materials and Methods

Four hundred and seventy-two adult men from the Veterans Affairs Medical Center (VAMC) at Dallas were recruited into a cross-sectional study designed to quantify risk factors for cardiovascular disease [14]. Several individuals that were enrolled had stable hypertension, a few (9% of total) also had type 2 diabetes mellitus treated with hypoglycemic agents consisting mostly of metformin and/or glyburide, and very few subjects (4%) had history of coronary heart disease (CHD). For the current analyses, men with type 2 diabetes mellitus were excluded. Data from 428 subjects was analyzed, and summaries are shown in Table 1 and Figures 2, 3, and 4. 

The racial composition of enrolled individuals was representative of research participants at the VAMC; that is, 72% were non-Hispanic White, 21% were non-Hispanic Black, 6% were Hispanic, and 1% comprised other groups. Subjects were seen in the clinical research unit, and they had clinical assessment and anthropometry. Fasting blood was drawn for measurement of plasma lipids, lipoproteins, glucose, hemoglobin A1c, apolipoprotein B, and adipokines. 

All study volunteers gave written informed consent to participate in the study that had been approved by the Institutional Research Board for Investigation in Humans.

## 3. Laboratory Measurements

Plasma total cholesterol, triglyceride, lipoprotein cholesterol, and apolipoprotein B were measured as previously described [14]. Levels of plasma insulin, leptin, and total adiponectin were measured by radioimmunoassay as detailed before [15]. 

## 4. Biostatistics

Data are summarized as means + SD or medians (interquartiles (IQ)), and comparisons of means were done by analysis of variance (ANOVA) with the Bonferroni adjustments for multiplicity of testing as needed. Some variables were positively skewed and were log transformed before parametric analyses (triglyceride, leptin, adiponectin, and HOMA2-IR). The Kruskal-Wallis rank test was carried out for comparisons of mean rank adiponectin/leptin ratios as a function of a number of risk factors for metabolic syndrome. Adiponectin and the ratio of adiponectin to leptin were also employed to create dichotomous groups of insulin resistance and dyslipidemia risk factors. The cut points were the median for adiponectin as previously detailed [13] and the median for the adiponectin/leptin ratios for each waist girth category. Accordingly, waist girth category <90 cm had a median ratio of 3.7, waist girth 90–101 cm had a median ratio of 1.39, and waist girth >90 cm category had a median ratio of .69. An SAS version of Stat View was employed. HOMA2-IR calculator described by Levy et al. [16] was used. 

## 5. Results

### 5.1. Characteristics of Study Subgroups

Men were subgrouped according to waist girth because this anthropometric measure is recommended for assessment of metabolic risk imparted by central obesity [1–3]. The waist girth cut points coincide with BMI cut points for nonobese (BMI <25 kg/m^2^ or waist girth <90 cm), overweight (BMI 25 to 29.9 kg/m^2^ or waist girth 90 to 101 cm), and obese men (BMI ≥30 kg/m^2^ or waist girth ≥102 cm) [17]. Accordingly, 13% of the men were nonobese, 34% were overweight, and 53% were obese. The men were of similar age across the waist girth subgroups (Table 1).

Several measures of insulin resistance were different among the subgroups. First, overweight and obese men had higher fasting glucose concentrations and tended to be more insulin resistant compared to the nonobese group as shown by the HOMA2-IR levels (Table 1). Overweight and obese men had a lower insulin sensitivity (HOMA2%S) compared with nonobese, but there were no significant differences in the steady-state beta-cell function estimated by HOMA2%*β*. Levels of hemoglobin A1c were similar among the subgroups. 

Overweight and obese men had higher triglyceride levels, reduced HDL cholesterol, and increased apolipoprotein B levels. However, levels of non-HDL cholesterol (i.e., VLDL + LDL) were not significantly different.

Nonobese men had lower blood pressure than overweight or obese men. The prevalence of hypertension (≥130/85 Hgmm or stable on antihypertensive medication) was high among the three subgroups; smoking prevalence was relatively low (Table 1). 

### 5.2. Interindividual Variation in Adipokines

**Figure 1:**
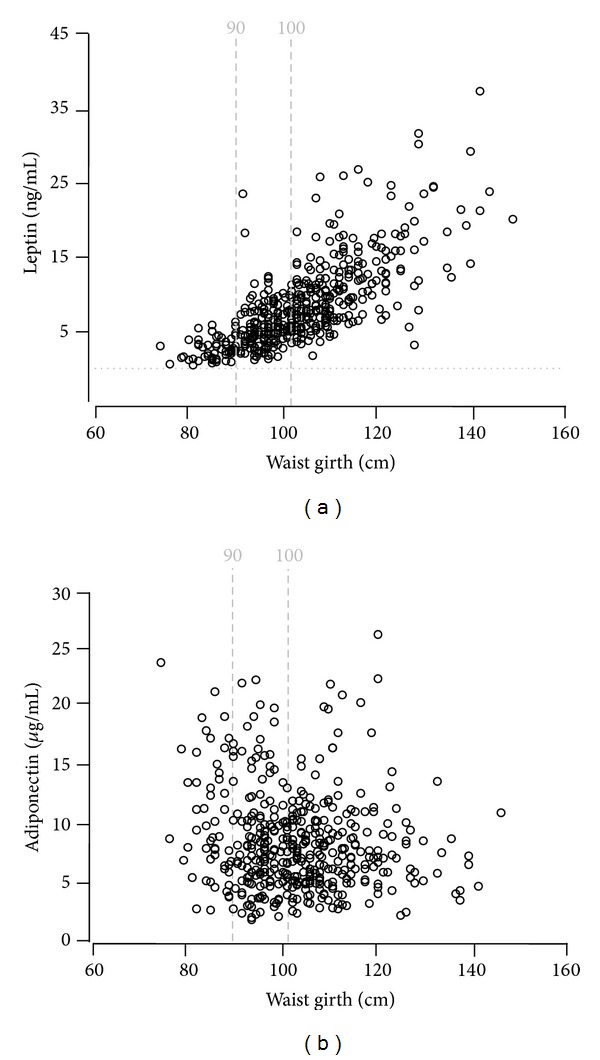
Scattered plots of leptin (a) and adiponectin (b) as a function of waist girth. There was a trend for a linear association of leptin to waist girth (*R* ^2^ = .56; *P* < .001). Subjects with waist girths below 90 cm consistently had the lowest leptin levels. More interindividual variations were noted in the leptin levels of subjects in waist girth in the range of 90 to 101 cm. In contrast to leptin, adiponectin was not linearly associated with waist girth (b). There was a striking interindividual variation in adiponectin levels at each waist girth subcategory, that is, nonobese, overweight, and obese.

Scattered plots of leptin versus waist girth (Figure 1(a)) and adiponectin versus waist girth (Figure 1(b)) were examined. There was a linear association of leptin to waist girth (Ln leptin = –19.267 + 4.569 (waist girth); *R* ^2^ = .56; *P* < .001). Lean men consistently had the lowest leptin levels (<5 ng/mL). More interindividual variations were noted in the leptin levels of overweight and obese men. In contrast to leptin, adiponectin was not linearly associated with waist girth (Figure 1(b)). Instead, there was a striking interindividual variation in adiponectin levels ranging from very low to high levels at each waist girth subcategory (nonobese, overweight, and obese individuals).

### 5.3. Adipokine Ratios and Metabolic Risk Factors

Levels of plasma triglyceride, HDL cholesterol, ratio of triglyceride/HDL cholesterol (C), HOMA2-IR, and systolic blood pressure were examined according to cut-off points for adiponectin (<6.5 ng/mL designated as low levels of adiponectin and ≥6.5 ng/mL designated as high levels) and according to adiponectin/leptin categories. The cut-off point for the ratio of adiponectin/leptin was the median ratio for each waist girth category as indicated in the statistical section. 

**Figure 2:**
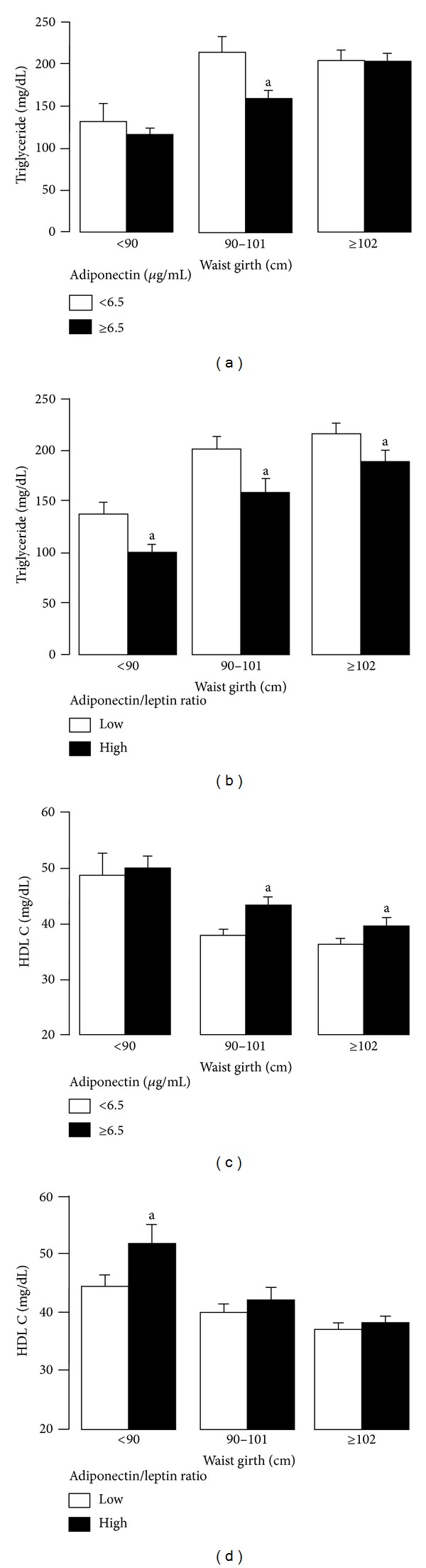
Levels of plasma triglyceride in nonobese (waist girth <90 cm), overweight (waist girth 90 to 101 cm), and obese (≥102 cm) grouped according to low (<6.5 ng/mL) or high (≥6.5 ng/mL) adiponectin. Overweight men with high adiponectin levels have significantly (^a^ *P* < .02) lower levels of plasma triglyceride compared with those with low adiponectin levels (a). However, after adjustment of adiponectin levels for leptin (ratio of adiponectin/leptin), levels of plasma triglycerides were significantly (^a^ *P* < .02) lower in those with a high ratio of adiponectin/leptin, regardless of waist category (nonobese, overweight, or obese) (b). Similar analyses are shown for HDL C. Levels of plasma HDL C were significantly (^a^ *P* < .02) higher in overweight and obese men having a high adiponectin level (c). However, HDL cholesterol levels were significantly higher (^a^ *P* < .02) in nonobese men after grouping the subjects according to a low or high ratio of adiponectin/leptin (d).

Levels of plasma triglyceride in nonobese, overweight, and obese men grouped according to low or high adiponectin levels were compared (Figure 2(a)). Overweight men with high adiponectin levels had significantly lower levels of plasma triglyceride (^a^ *P* < .02) compared with those men with low adiponectin levels. Levels of plasma triglyceride for each obesity category also were compared between men with low or high ratios of adiponectin/leptin (Figure 2(b)). Levels of plasma triglycerides were significantly lower (^a^ *P* < .02) in individuals with a high ratio of adiponectin/leptin, regardless of waist category (nonobese, overweight, or obese). 

HDL cholesterol (C) levels were significantly higher in overweight and obese men (^a^ *P* < .02) that had a high level of adiponectin compared with those that had a low level of (Figure 2(c)). However, HDL cholesterol levels were significantly higher in nonobese men (^a^ *P* < .02) after grouping the subjects according to a low or high ratio of adiponectin/leptin (Figure 2(d)).

**Figure 3:**
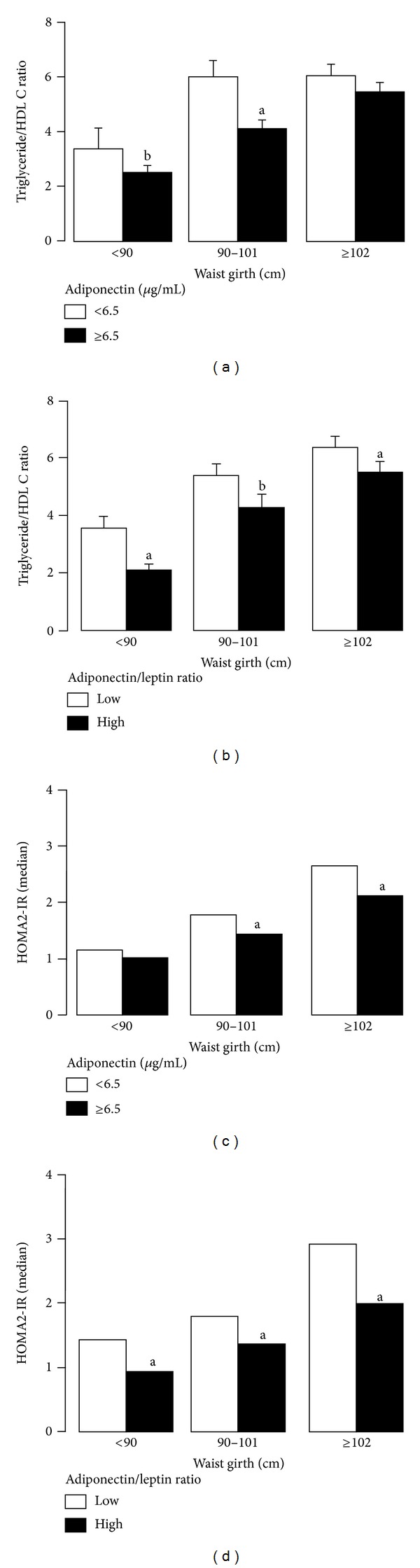
Ratio of triglyceride to HDL cholesterol (C) is lower in nonobese (^b^ *P* < .05) and overweight (^a^ *P* < .02) men who have a high level of plasma adiponectin (a). In contrast, the ratio of plasma triglyceride to HDL C is lower in nonobese, overweight, and obese subjects with a high ratio of adiponectin/leptin compared to those with a low ratio of adiponectin to leptin (b). HOMA2-IR levels are significantly higher (^a^ *P* < .02) in overweight and obese men with a high level of adiponectin compared with those with a low level of adiponectin (c). However, the HOMA2-IR levels are significantly lower in nonobese, overweight, and obese men who have a high ratio of adiponectin to leptin compared with those that have a low ratio (d).

Ratio of triglyceride/HDL cholesterol (C) was lower in nonobese (^b^ *P* < .05) and overweight (^a^ *P* < .02) men who had a high level of plasma adiponectin (Figure 3(a)). In contrast, the ratio of plasma triglyceride/HDL C was lower (^a^ *P* < .02) in nonobese, overweight, and obese subjects with a high ratio of adiponectin/leptin compared to those with a low ratio of adiponectin/leptin (Figure 3(b)).

HOMA2-IR levels are significantly higher (^a^ *P* < .02) in overweight and obese men with a high level of adiponectin compared with those with a low level of adiponectin (Figure 3(a)). However, the HOMA2-IR levels are significantly lower in nonobese, overweight, and obese men who had a high ratio of adiponectin/leptin compared with those that have a low ratio (Figure 3(b)).

**Figure 4:**
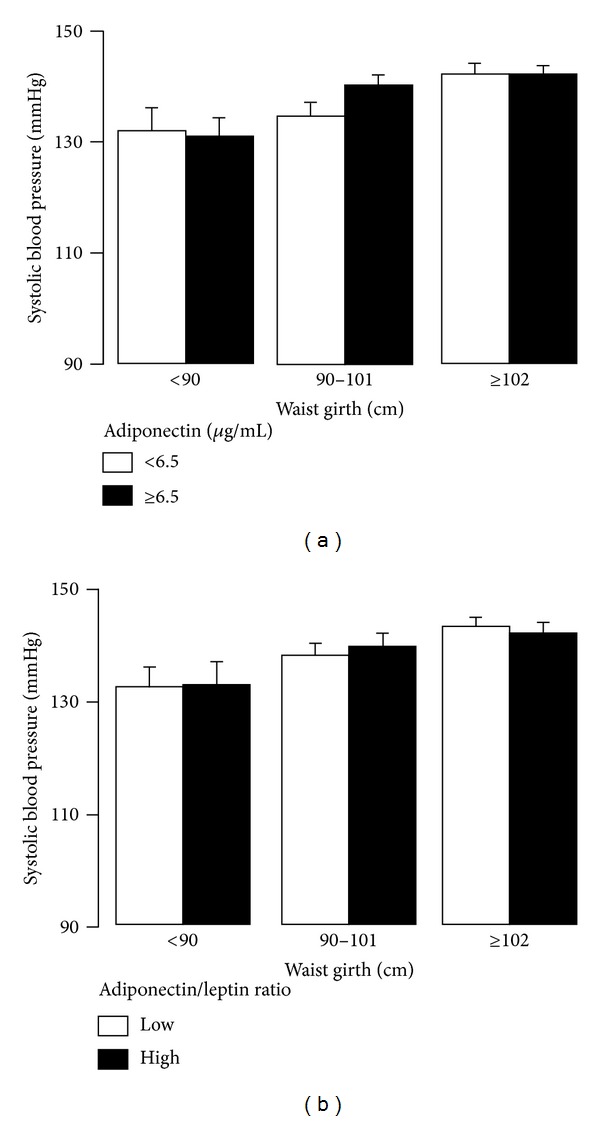
Systolic blood pressure in nonobese, overweight, and obese men was similar in those with low and those with high level of adiponectin (a). There were no significant differences in systolic blood pressure when the men were subgrouped according to the ratio of adiponectin/leptin (b).

Systolic blood pressure in nonobese, overweight, and obese men was similar in those with low and those with high level of adiponectin (Figure 4(a)) and in those with a high or low ratio of adiponectin/leptin (Figure 4(b)). 

**Figure 5:**
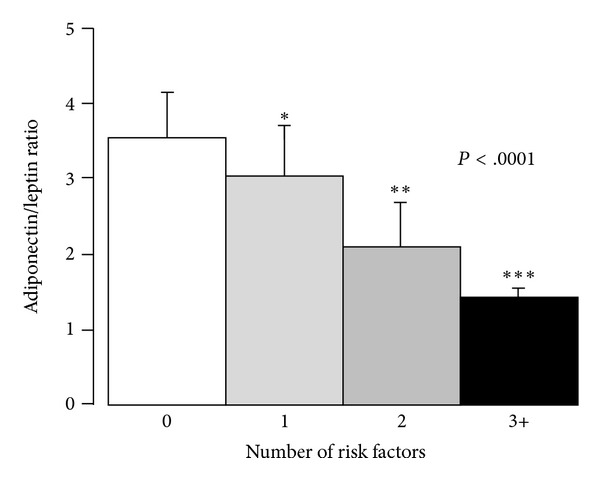
Plot of ratios of adiponectin/leptin versus number of risk factors. Subjects with ≥1 risk factors have a lower ratio than those without any risk factor. In addition, men with 2 risk factors have a significantly lower ratio than those with 1 risk, and those with ≥3 risks have significantly lower ratio than those with 1 or 2 risks.

The ratio of adiponectin/leptin in subjects with and without risks for metabolic syndrome that excluded waist girth was also examined using the Kruskal-Wallis rank test (Figure 5). Subjects with ≥1 risk factors have a lower ratio than those without any risk factor. In addition, men with 2 risk factors have a significantly lower ratio than those with 1 risk, and those with ≥3 risks have significantly lower ratio than those with 1 or 2 risks.

## 6. Discussion

This study examined the relation of adiponectin levels alone or normalized by leptin to metabolic risk factors for cardiovascular disease including markers of atherogenic dyslipidemia (levels of plasma triglyceride and HDL cholesterol and ratios of plasma triglyceride/HDL cholesterol) and insulin resistance (levels of HOMA2-IR) in men. The key observations made in this study were that high levels of adiponectin were associated with lower plasma triglycerides, higher HDL cholesterol, reduced ratios of triglyceride/HDL cholesterol, and reduced HOMA2-IR compared to lower levels of adiponectin in men regardless of waist girth. Normalizing adiponectin levels by leptin enhanced the associations of adiponectin to the metabolic risk factors. That is, subjects with a high ratio of adiponectin/leptin had lower triglycerides and triglyceride/HDL cholesterol ratios and higher HDL cholesterol and insulin sensitivity than those with low ratios of adiponectin/leptin regardless of waist girth. Thus, the adiponectin/leptin ratio was a useful index for identification of overweight and obese subjects with lower susceptibility to metabolic risk compared to individuals with a higher susceptibility. 

Both adiponectin and leptin have been implicated in the causation of dyslipidemia and insulin resistance. For example, leptin deficiency is associated with hypertriglyceridemia, low HDL C, and low insulin sensitivity in cases of acquired or congenital lipodystrophies [18]; leptin therapy reverses the metabolic dysfunction [19]. But in obese subjects, leptin does not have an effect [20]. Instead, leptin correlates with fat mass and it can be viewed as a biomarker of fat cell mass. Plasma levels of leptin generally reflect secretion rates by subcutaneous adipose tissue, principally by large adipocytes [9]. Omental fat also secretes leptin, but the subcutaneous fat is thought to be a major source of leptin. This hormone is known to modulate energy homeostasis through its action on hypothalamic receptors where it inhibits appetite [21]. Mutations in the human leptin gene are associated with hypogonadism and morbid obesity [22], and mutations in the human leptin receptor gene causes obesity and pituitary dysfunction [23]. Leptin replacement in obese subjects with leptin deficiency reverses the metabolic consequences of the deficiency in the hypothalamus [24].

In the current study, the linear association between plasma levels of leptin and waist girth provided a rationale for using leptin as a surrogate of fat mass. Moreover, overweight and obese men showed considerable interindividual variation in leptin levels despite the high correlation with waist girth. The interindividual variation also suggested that leptin levels are probably a more specific measure of fat mass than waist girth. For these reasons, leptin was used to normalize levels of adiponectin.

In contrast to leptin levels, plasma levels of adiponectin were not strongly correlated with waist girth. Others have shown an inverse association of adiponectin with total body fat [25]. However, the current study population showed a marked heterogeneity in plasma adiponectin level and this proved to be instructive. At any waist girth category, it was clear that there were men with high and low adiponectin levels. Thus, two questions could be addressed readily: (1) are there differences in levels of metabolic risk factors between men with high and low adiponectin levels regardless of waist girth? (2) If adiponectin levels are “normalized” for leptin levels, are the differences in metabolic risk factors better defined by the ratio of adiponectin/leptin than adiponectin alone?

The levels of plasma triglyceride were lower in men with a high adiponectin than in those with a low adiponectin. In contrast, HDL C, levels were higher in men who had high adiponectin compared with those with low adiponectin. Similarly, low ratios of plasma triglyceride/HDL C were lower in men with high adiponectin compared with those with low adiponectin. After normalizing adiponectin levels by leptin, the impact of the ratio of adiponectin/leptin on triglyceride, HDL C, and the ratio of triglyceride to HDL C was more apparent. The ratio segregated overweight and obese subjects into those with a relatively healthier metabolic profile and those with a higher-risk profile. Still the average plasma triglyceride levels of overweight and obese men with a high ratio of adiponectin/leptin were somewhat higher than the cut point of at-risk triglyceride (150 mg/dL). Perhaps a higher adiponectin/leptin ratio is needed to optimize triglyceride levels.

In contrast to leptin, adiponectin levels may be indicative of a protective effect of the adipokine on triglyceride metabolism even in the presence of excess body fat. Studies in animals suggest that adiponectin reduces levels of plasma triglyceride by increasing VLDL-triglyceride hydrolysis mediated by lipoprotein lipase [26]. Transgenic mice overexpressing adiponectin show a reduction in plasma triglycerides compared to wildtype [27–29] while adiponectin knockout mice have increased plasma triglycerides. These data suggest that adiponectin has a direct effect on triglyceride hydrolysis [30]. Other studies also suggest that adiponectin is an insulin sensitizer and it could exert a hypotriglyceridemic effect under such conditions. The association of adiponectin with HDL cholesterol may result from its hypotriglyceridemic effect or it could be the result of the effect of adiponectin on either apo A-I fractional catabolic rate [31, 32] or a direct effect of adiponectin on hepatic lipase [33, 34]. 

In the current study, high adiponectin levels also were generally associated with higher insulin sensitivity measured by HOMA2-IR than low adiponectin levels. This effect is supportive of the view that adiponectin is an insulin sensitizer [35, 36]. As such the adipokine can modulate the metabolism of triglycerides, HDL, and glucose. The exact mechanisms of the insulin sensitizing effect of adiponectin are not clearly understood. But in this study, it was clear that adiponectin and, more specifically, the ratio of adiponectin/leptin were a good indicator of insulin sensitivity in overweight and obese men. 

The adiponectin/leptin ratio also was shown to decrease with the increasing number of metabolic risk factors for cardiovascular disease (Figure 5). This ratio may be useful to identify subjects susceptible to metabolic risk, and adiponectin/leptin ratios may reflect the functionality of adipose tissue. Accordingly, two metabolic phenotypes were identified in overweight and obese subjects in the current study. It has been suggested that subjects are heterogeneous in the prevalence of metabolic risk factors. Some nonobese subjects have “metabolic obesity” [37]. Other investigators have identified obese subjects with a low prevalence of metabolic alterations which they have designated as “metabolically healthy” or “unhealthy” [38–40]. In the current study, the individuals with the high ratio of adiponectin/leptin had less dyslipidemia and insulin resistance than those with the low ratio, but they were not free of other risk factors. The ratio, however, suggested that adiponectin levels relative to leptin may be indices of susceptibility to metabolic risk. 

## 7. Conclusion

The current study shows that overweight and obese individuals with a high ratio of adiponectin/leptin have lower levels of plasma triglyceride, triglyceride/HDL C ratios and higher insulin sensitivity than those with lower adiponectin/leptin ratios. It is very likely that adiponectin contributes to the regulation of plasma triglyceride levels. The data also suggests that interindividual variation in dyslipidemia and insulin sensitivity is associated with the ratio of the adipokines. The study provides supportive evidence for the contention that some overweight and obese subjects have a better adipokine profile than others and that metabolic heterogeneity among overweight and obese subjects may depend on the ability of adipocytes to maintain secretion of the adipokines as the cells become filled with triglyceride. 

## Figures and Tables

**Table 1:** Subject demography and baseline characteristics.

	Nonobese	Overweight	Obese
Waist girth category	<90 cm	90–101 cm	≥102 cm
Number of men (% of total)	55 (13)	147 (34)	226 (53)
Age (±SD) (years)	53.0 (8.9)	54.1 (10.2)	55.4 (9.6)
Body mass index (kg/m^2^)	24.1 (2.3)	27.9 (2.5)^b^	33.6 (4.1)^a^
Waist girth (cm)	85.3 (3.6)	95.9 (2.9)^b^	112.8 (9.3)^a^
Systolic blood pressure (Hg mm)	132.1 (17.9)^c^	138.3 (16.7)	142.5 (16.7)
Diastolic blood pressure (Hg mm)	79.1 (12.9)^c^	82.3 (9.7)	83.0 (8.9)
Hemoglobin A1c (%), Median (IQ)*	5.4 (.4)	5.4 (.5)	5.5 (.6)
Glucose (mg/dL)	93.3 (9.3)^d^	94.9 (10.0)	97.0 (11.3)
HOMA2-IR median (IQ)	1.16 (.98)	1.57 (.77)	2.13 (1.53)^a^
HOMA2%*β*, median (IQ)	97.5 (51.9)	119.8 (47.4)	139.9 (70.6)
HOMA2%S, median (IQ)	86.5 (101.7)	63.7 (33.3)^b^	46.9 (36.4)^a^
Adiponectin *μ*g/mL, median (IQ)	8.9 (87.6)^c^	7.7 (4.8)	7.5 (4.7)
Leptin ng/mL, median (IQ)	2.3 (2.2)	5.2 (3.9)^b^	10.4 (7.5)^a^
Triglyceride, median (IQ)	110 (68)	153 (104)^b^	186 (92)^a^
HDL cholesterol mg/dL	47 (15)	39 (15)^b^	37 (9)^a^
Non-HDL cholesterol mg/dL	147 (44)	166 (43)	165 (47)
Total Apo B mg/dL	116 (29)	126 (28)^b^	129 (26)^a^
Hypertension (%)	52.6	75.5	88.8
Smokers (%)	.0	3.7	1.5

^a^Significantly different from non-obese and overweight (*P* < .02); ^b^significantly different from non-obese (*P* < .02); ^c^significantly different from overweight and from obese (*P* < .02); ^d^significantly different from obese (*P* < .02); *IQ: interquartile.
